# Adsorption of lead and chromium ions from electroplating wastewater using plantain stalk modified by amorphous alumina developed from waste cans

**DOI:** 10.1038/s41598-024-56183-2

**Published:** 2024-03-13

**Authors:** E. O. Ajala, M. O. Aliyu, M. A. Ajala, G. Mamba, A. M. Ndana, T. S. Olatunde

**Affiliations:** 1 Department of Chemical Engineering, University of Ilorin, Ilorin, Nigeria; 2 Institute for Nanotechnology and Water Sustainability, College of Science, Engineering, and Technology, University of South Africa, Florida, Johannesburg, 1709 South Africa; 3 Department of Chemical Engineering, Federal Polytechnic, Bida, Nigeria; 4 Department of Food Science and Technology, Federal University of Technology, Akure, Nigeria

**Keywords:** Chemistry, Engineering, Materials science

## Abstract

Waste beneficiation is key to environmental protection and the realisation of a circular economy. Herein, amorphous alumina (a-Al_2_O_3_) derived from aluminium waste cans (AWC) was used to modify plantain stalk as an adsorbent for sequestration of lead (II) and chromium (VI) ions from electroplating wastewater. Raw plantain-stalk (RPS) and amorphous-alumina modified plantain stalk (APS) developed as adsorbents were characterised using various equipment such as x-ray diffraction (XRD), thermogravimetric analysis (TGA), scanning electron microscopy (SEM), Fourier Transform Infrared Spectroscopy (FTIR), and Brunauer–Emmett–Teller (BET). The FTIR revealed that the adsorbents are rich in functional groups that could promote the adsorption process which includes carboxyl, hydroxyl, and aliphatic groups. Also, the BET analysis showed a substantial increase in the surface area of APS (174.448 m^2^/g) compared to that of RPS (40.531 m^2^/g) which could be due to the effect of modification by the a-Al_2_O_3_. The batch adsorption studies revealed that the APS achieved 99.38% and 98.33% removal of Cr(VI) and Pb(II), respectively, which is superior to RPS adsorption efficiency. Also, the estimated and experimental data for the APS compared well under all the kinetic models studied with R^2^ > 0.88. This suggested that chemisorption is the most plausible adsorption mechanism of Cr(VI) and Pb(II) onto the APS. Further analysis showed that the Cr(VI) and Pb(II) adsorption followed the Langmuir model with the R_L_ value of 0.038 and 0.999, respectively, which indicated that the two metal ions were effectively adsorbed onto the APS. Therefore, this work demonstrated that the modification of plantain-stalk with amorphous-alumina derived from AWC enhanced the characteristics of the APS and favoured its adsorption of the selected heavy metals.

## Introduction

Rapid urban development, growing populations, and expansion of industries cause a high generation of wastewater that is eventually disposed into freshwater bodies^1^. Pollutants in untreated or poorly treated wastewater effluent, such as heavy metals, dyes, phenols, inorganic anions, and pesticides, are harmful to human and aquatic lives^2–4^. Typically, electroplating industry discharges large volume of wastewater laden with high concentrations of hazardous heavy metals such as Cu, Fe, Ni, Cr, Cd, and Pb, among other pollutants. These heavy metals are non-degradable, highly toxic and carcinogenic in concentrations above the permissible limits as recommended by the Environmental Protection Agency of the United States and World Health Organization^5^. Among the chromium species, Cr(VI) is the most prevalent oxidation state which is highly soluble, easily adsorbed and accumulated in human organs such as the kidney, liver, and stomach, causing adverse health effects^6^. Similarly, Pb(II) is another toxic heavy metal even when present in trace quantities, and when consumed or inhaled it could seriously harm the kidneys, and cause neurological and reproductive systems disorder^7^. Therefore, these heavy metals must be removed from the wastewater before discharging it to the environment.

Several technologies exist for the remediation of heavy metals and other impurities from industrial wastewater which include electrochemical methods, coagulation/flocculation, ion flotation, photocatalysis, ion exchange, chemical precipitation, membrane separation and adsorption^8^. However, the adsorption techniques have been considered as the most effective methods for the removal of heavy metals due to their high efficiency, low cost-effectiveness, easy to use and their reusability^5^. Specifically, the adsorption process has been reported as the most popular method for treating wastewater containing lead, chromium and other heavy metals due to the low production of by-products^9–11^. When choosing an adsorbent to treat wastewater, two important variables must be considered, which are technical applicability and cost-effectiveness. Organic materials, industrial waste, agricultural waste, and biological waste are all forms of materials that are regarded as potential adsorbents as they fulfil the aforementioned important variables.

Numerous investigations have been conducted on the potential use of agricultural wastes as efficient adsorbents for the removal of metal ions from aqueous solutions including wastewater. Such wastes include sugarcane bagasse^12^, plantain peel^13^, orange and potato peels^14^, plantain stalk^15^ and coconut coir^7^. Agricultural by-products are easily accessible and frequently cause problems with trash disposal. Their application as adsorbents employs the concept of waste beneficiation and circular economy into practice. In addition to their abundance and environmental friendliness, agricultural by-product-derived adsorbents are more cost-effective than traditional adsorbents like activated carbon. Using agricultural by-products for wastewater treatment also eliminates the need for complicated regeneration processes^16^.

A readily available lignocellulosic agricultural waste with a high carbon content is plantain/banana stalk^17^. However, large quantities of the stalks (as waste products) are typically dumped in ponds and rivers, where they slowly degrade and produce gases like methane that harm the local ecology and spread a putrid smell^17^. Despite the numerous investigations on the remediation of heavy metals contaminations, the majority of research focused on the purification of single metal-contaminated water. While others used spiked aqueous solutions to study the effectiveness of the adsorbents in removing heavy metals. However, in real industrial effluent, the heavy metals co-exist and interact amongst themselves and other wastewater constituents. Consequently, there is an urgent need to create innovative products and technology for the concurrent elimination of these contaminants from industrial wastewater. One of the approaches to achieve this is the modification of lignocellulosic agricultural waste with metal oxides such as TiO_2_, Ag_2_O, ZnO, CuO, and Al_2_O_3_^4^. The Al_2_O_3_ having numerous characteristics such as large surface area, great physicochemical properties, high dielectric strength, and thermal stability, is an adsorbent agent for wastewater treatment and a suitable adsorbent support^18–21^.

Meanwhile, sustainable development through circular economy, alternative sources, and enforcement of environmental standards have necessitated the conversion of municipal wastes, such as AWC, for the production of Al_2_O_3_^22^. Recycling AWC provides environmental and economic benefits as it saves precious natural resources, energy, time, and money^23^. Worthy of note is that the recycling of AWC is a process of dual benefits; reduction of the accumulated amount of waste and a superficial way for synthesizing new product that has significant applications in wastewater treatment^24^. Hence, this work explored the development of an adsorbent from plantain stalk modified with a-Al_2_O_3_ (APS) produced from AWC for the removal of Pb(II) and Cr(VI) ions in electroplating wastewater, which is a novel research study. The Al_2_O_3_ is a material with excellent characteristics such as high porosity, low density, and high specific surface area. Due to its nanopores and extraordinary networks, Al_2_O_3_ is a potential material in porous membranes, catalyst support, and adsorbent production^25^. Some researchers have reported the use of Al_2_O_3_, either as a unit or in combination with other materials, to remove the heavy metal ions in wastewater. The Al_2_O_3_/Pd(NO_3_)_2_/zeolite composite film was developed for the adsorption of carbon monoxide gas and attained 97% removal^26^. Banana peel-activated carbon coated with Al_2_O_3_-chitosan was fabricated as an adsorbent for the removal of Pb(II) (99.9% removal), and Cd(II) (99.9% removal) ions from wastewater^27^. Also, the removal of congo red dye from an aqueous solution using Al_2_O_3_/ZnO composite was achieved by the adsorption process at 97.35% removal^28^.

Therefore, this study aims to investigate the influence of modifying the plantain stalk with waste aluminium cans derived a-Al_2_O_3_ as an adsorbent for the remediation of electroplating wastewater laden with Pb(II) and Cr(VI) ions. The composite adsorbent and other precursors were characterised for their physical and chemical properties using various equipment such as SEM, XRD, BET, and FTIR. The influence of adsorption parameters such as adsorbent dosage, contact time, and temperature were investigated to establish the optimal conditions for the sequestration of Pb(II) and Cr(VI) ions. Ultimately, the adsorption behaviour and mechanisms were investigated and outlined in detail. The reusability and regenerative capacity of the adsorbent were also investigated in this study.

## Experimental method

### Materials and reagents

**Figure 1:**
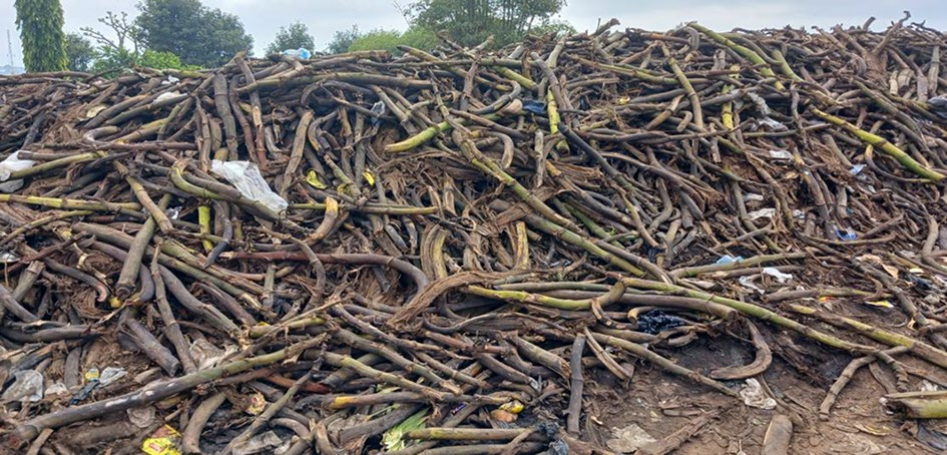
Dumping site of plantain stalk in *Ganmo* market, *Ganmo*, Kwara State, Nigeria.

The RPS were sourced from a dumping site shown in Fig. 1, located in *Ganmo* market, *Ganmo*, Kwara State, Nigeria. Meanwhile, sulphuric acid, 98.08%, hydrochloric acid, 37%, NaOH pellets, dimethyl sulfoxide (DMSO), and tetra ethyl-o- silicate (TEOS) were obtained from Loba Chemie Laboratory Reagents and Fine Chemicals. Ltd, Nigeria. The electroplating wastewater was collected from the Tatchrome Industry in Kaduna, Nigeria. All the chemicals used were of analytical grade and were deployed without any further purification.

### Preparation of raw plantain stalks and amorphous-alumina

The RPS was shredded into smaller sizes using a sharp stainless-steel knife. The sample was thoroughly washed with distilled water in a small plastic container to eradicate impurities. Then, the sample was spread on trays and sun-dried for seven days to completely remove the moisture content. This was followed by pulverizing the RPS sample using a 9FQ40 Hammer Mill and sieved with a 200 µm sieve to obtain a powder of uniform size. The RPS powder was then stored in air-tight polythene vials and kept in a desiccator for further studies.

To prepare the a-Al_2_O_3_, AWC were collected in a guest house along the University of Ilorin road, Ilorin, Kwara State. The AWC were shredded into smaller pieces using a pair of stainless-steel scissors. The colour film used to coat the AWC was removed by washing with 5 M of sulfuric acid. Subsequently, 10 g of the AWC sample was dissolved in 5 M HCl to obtain an AlCl_3_ solution, and the polymeric residue was discarded. The AlCl_3_ solution was added to 5 M NaOH to form an Al(OH)_3_ precipitate, which was separated from the supernatant using filtration. The Al(OH)_3_ precipitate was thoroughly washed using de-ionized water to remove NaCl impurities and dried in an oven at 200 °C for 12 h. Finally, the Al(OH)_3_ powder was calcined at 900 °C in a muffle furnace (Carbolite CWF1200/ UK) with a heating rate of 10 °C/min for 1 h to obtain stable a-Al_2_O_3_.

### Modification of RPS with a-Al_2_O_3_ to synthesize APS adsorbent

The modified method described by Herrera-Barros et al.^29^ was employed for the development of alumina-modified plantain-stalk (APS) adsorbent. The adsorbent was prepared by loading a-Al_2_O_3_ onto the RPS sample in a ratio of 1:1 (w/w) using dimethyl sulfoxide (DMSO) as an organic solvent. The RPS was measured into a 250 mL conical flask that contained DMSO solution to form a suspension after stirring for 24 h using a magnetic stirrer. Thereafter, tetra ethyl-o-silicate (TEOS) and a-Al_2_O_3_ were added to the suspension and allowed to age for 6 h. The prepared adsorbent was then washed with ethanol to remove soluble impurities, followed by de-ionised water until a pH of 7 was attained. Thereafter, the sample was oven-dried at 100 °C until constant weight was achieved and allowed to cool to room temperature. The sample was identified as APS adsorbent, stored in polythene, and kept in a desiccator to prevent external contaminants, moisture, and other adverse conditions.

### Characterisations of adsorbent and other samples

Thermal stability and behaviour of the RPS were carried out using a PerkinElmer TGA-4000 thermogravimetric analyzer (TGA). X-ray diffraction (Bruker AxSD8) was used to probe the crystallinity, phase composition, and purity of the RPS, a-Al_2_O_3,_ and APS samples. Fourier transform infrared spectroscopy (Shimadzu FTIR 8400S, Japan) analysis was employed to investigate the functional group present on the surface of the samples. A TESCAN MIRA3 Scanning Electronic Microscope (SEM) was used to study the surface morphologies of the samples. Brunauer–Emmett–Teller (BET, Quantachrome instrument version 11.03) was used to determine the pore size, volume, and surface area of the samples as well.

### Batch adsorption experiments

In the first instance, heavy metal ions present in the electroplating wastewater were measured using a TG 990 Atomic Absorption Spectrometer (AAS). Thereafter, the adsorptive capacity of the RPS and APS samples for the sequestration of Cr(VI) and Pb(II) ions in the electroplating wastewater was examined in a water bath shaker (WS-100 water bath shaker). Each of the RPS and APS samples (0.75 g) was measured into different 200 mL conical flasks containing 100 mL of the electroplating wastewater. The flask was shaken in the water bath shaker for 90 min at 200 rpm and 30 °C. The residual concentration of the heavy metal ions in the treated wastewater was determined using the AAS. The percentage removal of the metal ions and adsorption capacity were calculated using Eqs. (1) and (2), respectively.

1 $$\mathrm{\%}Removal=\frac{{C}_{0}-{C}_{e}}{{C}_{0}}\times 100$$

2 $${q}_{e}=\frac{v\left({C}_{0}-{C}_{e}\right)}{M}$$

where; v is the volume of the wastewater to be treated, ${C}_{0}$ is the starting concentration of the metal ions in the wastewater (mg/L), Ce is the equilibrium concentration of the metal ions in the wastewater (mg/L), M is the weight of the adsorbent (g), and q_e_ is the equilibrium adsorption capacity (mg/g).

The effect of adsorbent dosage (0.75, 1.0, 1.25, or 1.5 g), contact times (30, 60, 90, 120, 150, or 180 min), and temperatures (30, 45, 60, or 75) were thereafter investigated in a one-factor-at-a-time. Kinetics and isotherm models, as well as the thermodynamic parameters that control the adsorption process were also studied using established models and equations.

### Statement on the use of plant material

The use of agricultural waste (biomass) such as plantain stalk in this study is in accordance with relevant institutional, national, and international guidelines and legislation.

## Results and discussion

### Thermal stability of RPS

**Figure 2:**
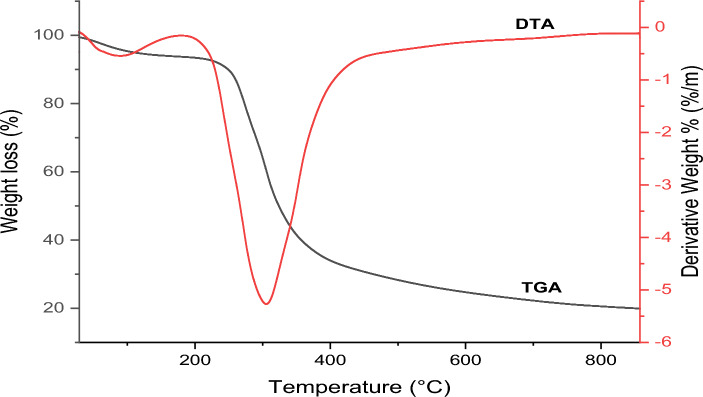
TGA and DTA curves of the RPS sample.

The thermal stability and behaviour of the RPS as obtained from the thermogravimetric analysis (TGA) are presented in Fig. 2. The TGA curve depicts that there was no weight loss at a temperature below 50 °C. However, three distinct phases of weight loss were noticed at advanced temperatures from the curve. An initial weight loss of roughly 6% was observed between 50 °C and 180 °C of temperature which was due to loss of moisture in the sample. The major weight loss of about 60% occurred between 180 °C and 450 °C of temperature, which could be attributed to the decomposition of hemicellulose, cellulose, and lignin. Generally, the lignin decomposes slowly for all plant fibres at a temperature ≥ 700 °C^30^, although a noticeable weight loss was observed for the RPS sample at a temperature of 450 °C. The observed percentage of weight loss at this temperature is comparable to the findings in the literature^31^.

The plot of Differential thermal analysis (DTA) for the RPS shows that the first deterioration or initial decomposition occurred at a temperature between 30 and 170 °C. This weight loss which is approximately 6% could be due to loss of moisture or fiber volatilisation and degradation^32^. Meanwhile, between 200 and 400 °C, there was a major decomposition with a maximum weight loss occurring at about 300 °C. Beyond 450 °C, the RPS appears thermally stable and thus, the temperature was considered as a basis for the adsorbent development.

### XRD and FTIR analysis

**Figure 3:**
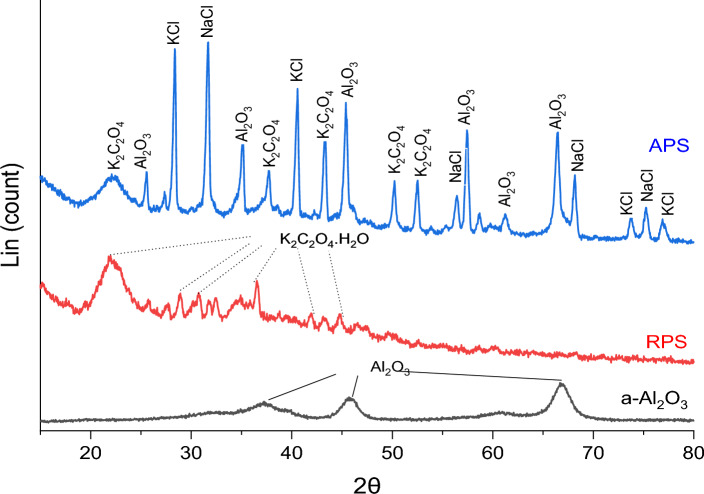
XRD patterns of a-Al_2_O_3,_ RPS, and APS samples.

Figure 3 displays the XRD patterns of RPS, a-Al_2_O_3_, and APS samples. The XRD analysis of the RPS indicates that its main crystalline phase component is potassium oxalate hydrate (K_2_C_2_O_4_.H_2_O). This finding is consistent with a similar result reported by Deng et al. (2020). In contrast, alumina exhibits broad peaks, indicating its amorphous nature. This observation aligns with the results reported in the literature^33,34^ where authors observed the formation of amorphous alumina at a calcination temperature of 900 °C. Upon impregnating RPS with a-Al_2_O_3_ to form APS adsorbent, the formation of spectra of both samples (RPS and a-Al_2_O_3_) were seen in the figure. However, distinct changes were also observed in the peaks, as some spectra of KCl and NaCl were also noticed, which could be the products of elements involved in the process. Noteworthy is the fact that KCl and NaCl are efficient in the removal of Cr(II) and other pollutants in the wastewater^35,36^.

**Figure 4:**
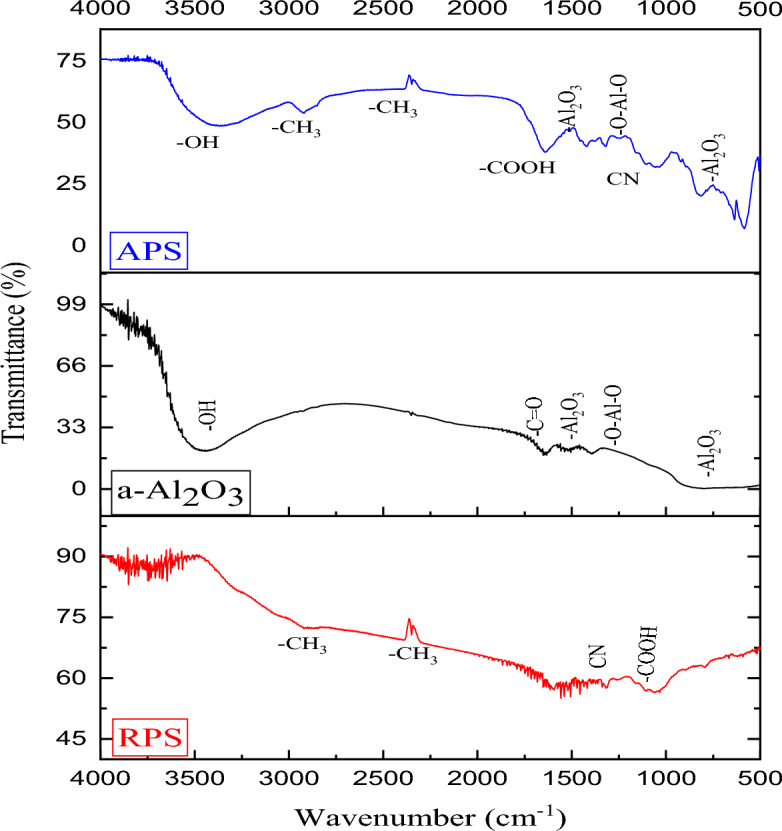
FTIR spectra of RPS, a-Al_2_O_3_, and APS.

Identification of the surface functional groups is important in understanding the active sites for the adsorption of the heavy metal species^37^. Therefore, Fig. 4 shows the functional groups of RPS, a-Al_2_O_3_, and APS in the form of FTIR spectra. The figure revealed that the samples are embellished with carboxyl, hydroxyl, aliphatic, and amine functional groups, which are good adsorptive characteristics. The spectra of a-Al_2_O_3_, as presented in the figure show bonds at 3385.75 and 1631.66 cm^−1^ which are the attributes of the stretch band of OH and C=O groups on the surface of the sample^38^. Also, the presence of a broad band at 675.46 cm^−1^ suggests the metal oxide bond stretching in the Al_2_O_3_ structure^39–42^. Also, the a-Al_2_O_3_ sample reveals the presence of peaks at 1521.16 cm^−1^ which may probably correspond to amorphous alumina^43^. However, when the RPS and a-Al_2_O_3_ samples were impregnated to form the APS adsorbent, the disappearance, shifting, and reduction (in intensities) of some peaks were noticed. The hydroxyl group (-OH stretch) was observed with the APS at 3400 cm^−1^, which is an extremely important functional group for effective adsorption and exchange with metal ions.

Meanwhile, the methyl group (-CH_3_) was observed in both RPS and APS at similar wavenumbers (2929.8 and 2850 cm^−1^). Furthermore, the –COOH peak was observed at 1400 cm^−1^ for RPS and shifted to 1550 cm^−1^ for APS. The presence of these peaks in the samples are due to the organic nature of the RPS. Also, both the APS and RPS show similar aromatic secondary amine (–CN stretch), which originate from the RPS as a chemical constituent of the material. Comparatively, FTIR peaks of the RPS, which are relatively broad spectra, became sharp peaks due to the incorporation of a-Al_2_O_3_. The presence of additional negatively charged groups on the surface of the APS could result in a relatively high adsorption rate^44^.

### Surface morphology and BET analyses of the samples

**Figure 5:**
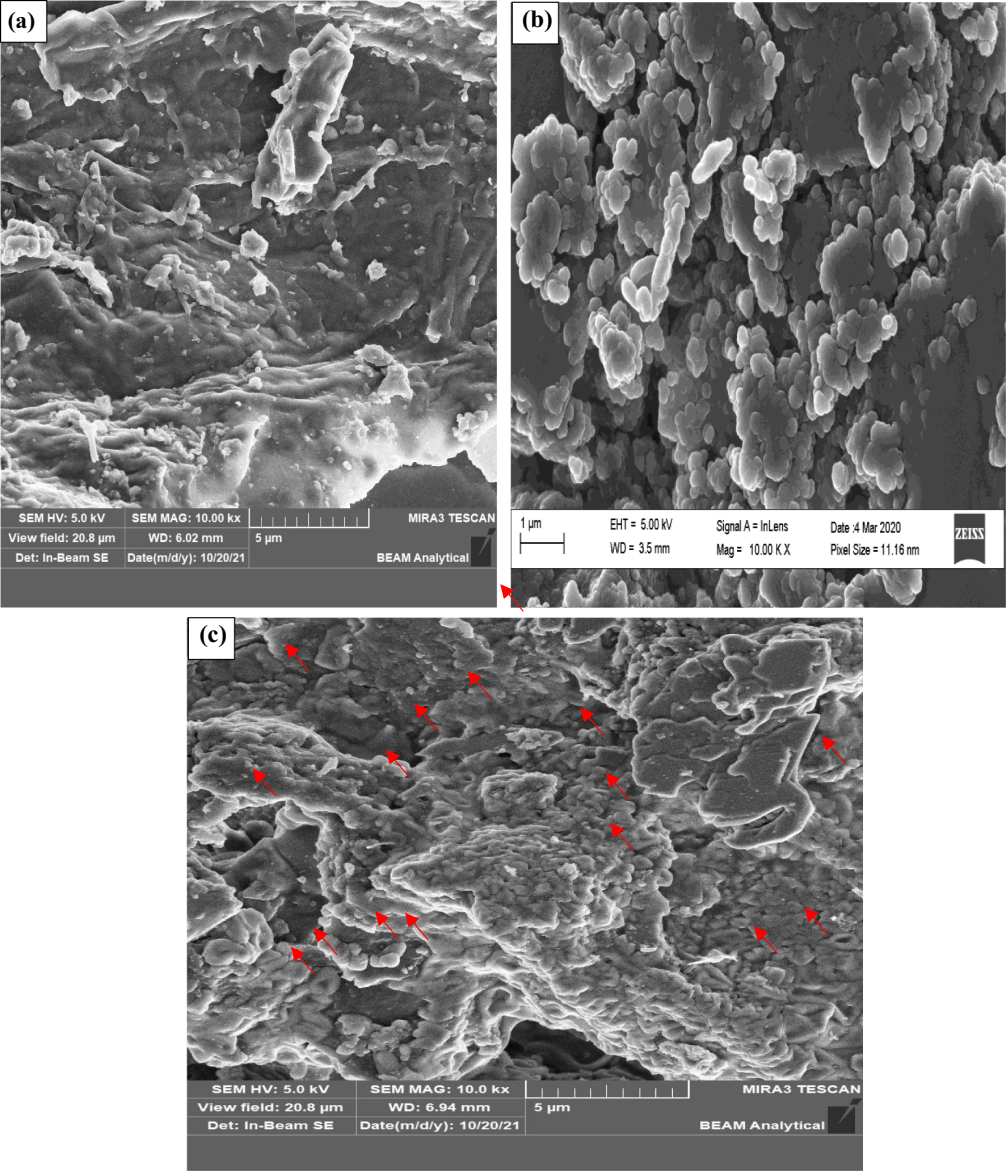
SEM images for (**a**) RPS, (**b**) a-Al_2_O_3_, and (**c**) APS.

The surface morphology of the RPS, a-Al_2_O_3_, and APS samples was probed using the SEM, and the images obtained are depicted in Fig. 5a–c, respectively. The micrograph of the RPS (Fig. 5a) shows a merged structure with the absence of porosity and a crinkled morphology, which are characteristic of any biomass^45^. This sample may not be efficient for the adsorption of heavy metals in the wastewater treatment process due to its non-porous and crinkle nature. The image of a-Al_2_O_3_ obtained, as shown in Fig. 5b presents nearly spherical particles with uneven surfaces. Also, the particles of the sample are relatively uniform in size and show a certain level of aggregation to form large clusters. This is consistent with the findings of Rabia et al.^46^ who reported that the Al_2_O_3_ image has uneven flakes with rough surfaces and tiny gaps (pore spaces) among the particles. Figure 5c indicates that upon modification of the RPS with a-Al_2_O_3_, the composite showed a morphology characteristic of the two materials. The image revealed that the a-Al_2_O_3_ particles are deposited as shown with red arrows, on the surface of the RPS.

The BET analysis of the RPS, a-Al_2_O_3,_ and APS samples are presented in Table 1. After RPS was activated with a-Al_2_O_3_, the results showed a considerable change in the surface area as the modified RPS (APS) has 174.448 m^2^/g compared to 40.531 m^2^/g and 79.05 m^2^/g for RPS and a-Al_2_O_3_, respectively. This increase in the surface area of the APS compared to the RPS was more than 4 times, which revealed the effect of the a-Al_2_O_3_ on the modification. The significant increase in the surface area of the APS is beneficial for the adsorption of heavy metal ions and may have promoted the transfer of heavy metal ions^45^. Also, the values of the pore volume and pore size of the samples showed that the APS values fall within the obtained values for both the RPS and a-Al_2_O_3_. This further justified the effect of the impregnation of RPS with the a-Al_2_O_3_ as there was a noticeable increase in the pore volume and pore size of APS (0.098 cc/g and 2.126 nm, respectively) compared to that of the RPS (0.023 cc/g and 0.544 nm, respectively). This could be due to the nature of a-Al_2_O_3_, as it has a high surface area. Thus, the incorporation of a-Al_2_O_3_ onto the surface of the plantain stalk can enhance its adsorption effectiveness by increasing the surface area of the adsorbent. Whilst also provides additional active sites for the sorption of the heavy metal ions (Pb(II) and Cr(VI) ions) in the wastewater^47^.

**Table 1:** Surface area and pores characteristics of RPS and APS.

Samples	Surface area (m^2^/g)	Pore volume (cc/g)	Pore size (nm)
RPS	40.531	0.0228	0.544
a-Al_2_O_3_	79.05	0.125	3.15
APS	174.448	0.098	2.128

### Adsorption experiments

The presence of some heavy metal ions was investigated in the electroplating wastewater before and after adsorption, and the results are presented in Table 2. These results were also compared with the WHO and U.S.EPA standards, as shown in Table 2. The process conditions of adsorbent dosage of 0.75 g, contact time of 90 min, temperature of 30 °C, and shaking speed of 200 rpm were considered for the RPS and APS in the preliminary study. The results of the wastewater before adsorption revealed that it was laden with heavy metal ions such as Pb (0.600 mg/L), Cr (39.660 mg/L), Ni (38.130 mg/L), Zn (3.650 mg/L), Fe (5.930 mg/L), Cu (2.060 mg/L) and Cd (0.044 mg/L). The concentrations of the metal ions obtained were beyond the permissible limit of the WHO and U.S. EPA standards. Hence, it is imperative to eliminate them from the wastewater before discharging the water into the environment. The preliminary investigation of the adsorption process using RPS and APS shows that Pb, Cr, Ni, Zn, Fe, Cu, and Cd metal ions concentration reduced in the wastewater to 0.160 and 0.050 mg/L, 18.370 and 11.610 mg/L, 16.860 and 27.760 mg/L, 3.020 and 1.450 mg/L. 4.500 and 0.140 mg/L, 1,250 and 0.200 mg/L, and 0.026 and 0.000 mg/L, respectively. These results revealed that the APS had a better adsorption performance compared to the RPS, resulting in a significant reduction in most of the metal ions concentrations except Ni (27.760 mg/L). The reason for the ineffectiveness of the APS for the sequestration of Ni in the wastewater could be due to its low affinity for the ions^48^. It could also be that the APS requires enhancement and reinforcement of the functional group through the pre-treatment/modification process, to increase the number of active sites^49^. The incorporation of the a-Al_2_O_3_, therefore, significantly improved the adsorption capacity of the APS for all the metal ions in the wastewater except the Ni ions. This could also be attributed to the presence of some functional groups, such as O–H, –CN, and –COOH, which have affinities for the metal ions. Therefore, further adsorption studies on the use of APS in this research were limited to the removal of Pb and Cr ions in the electroplating wastewater.

**Table 2:** Metal ions concentration in electroplating wastewater and the standards.

Heavy	Adsorption (mg/L)			WHO	U.S.EPA
Metals	Before	After		(mg/L)	(mg/L)
		RPS	APS		
Pb	0.600	0.160	0.050	0.010	0.050
Cr	39.660	18.370	11.610	0.050	0.050
Ni	38.130	16.860	27.760	0.020	0.100
Zn	3.650	3.020	1.450	3.000	2.000
Fe	5.930	4.500	0.140	0.300	2.000
Cu	2.060	1.250	0.200	1.000	0.500
Cd	0.044	0.026	0.000	0.003	0.010

#### Effect of adsorbent dosage

**Figure 6:**
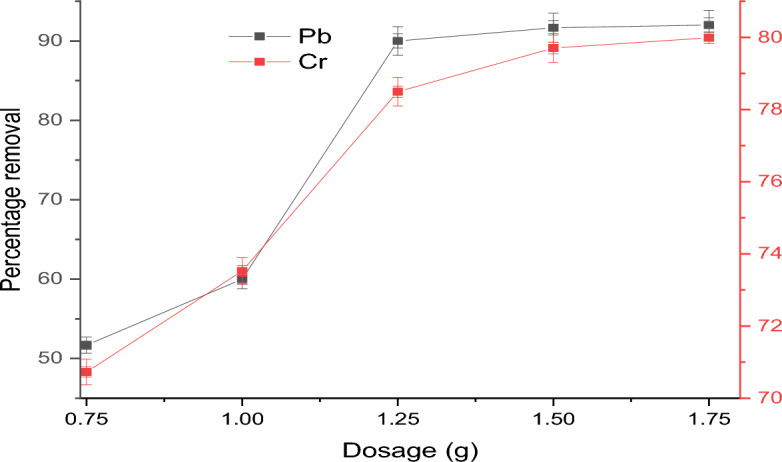
Effect of APS dosage on the removal of Pb(II) and Cr(VI) ions.

Figure 6 shows the effect of the adsorbent dosage of 0.75, 1.0, 1.25, 1.5, or 1.75 g on the removal of Pb(II) and Cr(VI) ions by the APS. The study was evaluated in a batch process while keeping all other parameters constant. The figure illustrates the impact of adsorbent dosage, where an increase in the dosage increases the percentage removal for both Pb(II) and Cr(VI) ions. The removal efficiency for Pb(II) ions increases from 52% to 92%, while that of Cr(VI) ions increases from 71% to 80%. At the optimum dosage of 1.5 g, the removal efficiency reaches its peak with 92% of Pb(II) ions and 80% of Cr(VI) ions being effectively removed. These findings can be attributed to the surface area of the adsorbent, which increases with higher dosages and resulting in an increased number of active sites for the removal of metal ions from the wastewater. This explanation aligns with the findings in the literature^50^. However, as the adsorbent dosage increased to 1.75 g, the percentage removal of both Pb(II) and Cr(VI) ions remained stagnant. This could be due to the exposure of a substantial amount of accessible binding sites to the small adsorbate in the medium which resulted in less per-gram adsorption. This prevented the higher adsorbent dosage of more than 1.5 g from being shielded from further adsorbing the heavy metals (Pb(II) and Cr(VI)) ions^4^. The huge adsorbent quantities could result in cell agglomeration and then a consequent decrease in intercellular distance. It also induced a ‘screen effect’ among a dense layer of cells, causing the ‘protection’ of binding positions from metal ions^51^. A similar trend was reported in the literature when flamboyant pod-activated carbon was used to remove Pb(II) and Cr(VI) ions from an aqueous solution^52^.

#### Effect of contact time

Similarly, the effect of contact time of 30, 60, 90, 120, 150, or 180 min was evaluated at the adsorbent dosage of 1.5 g while other parameters were kept constant. This study is necessary as the equilibrium contact time is important to establish how long it takes for the adsorption of the heavy metals to reach the saturation limit of the adsorbent.

**Figure 7:**
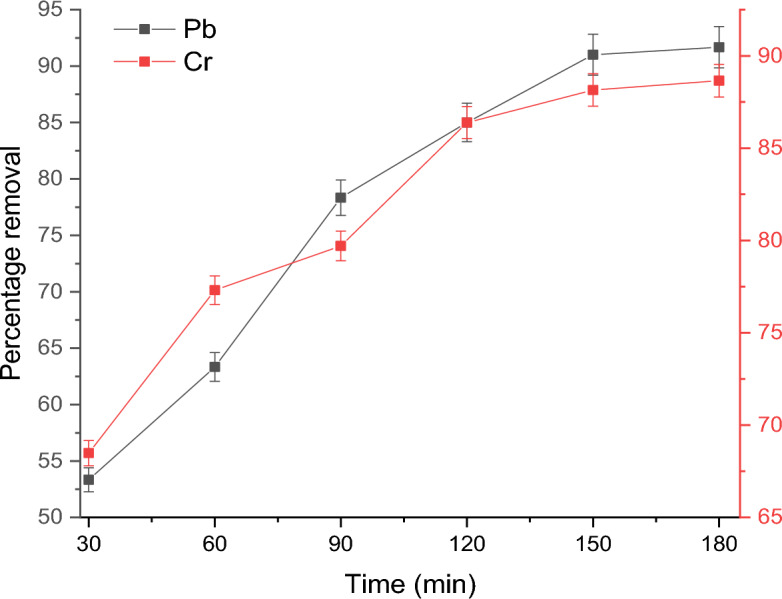
Effect of contact time on the removal of Pb(II) and Cr(VI) ions by the APS.

Figure 7 illustrates how the adsorption of the metal ions changed with increasing contact time up to 180 min. It can be seen that between 30 and 120 min of adsorbent-adsorbate contact time, there was noticeably immediate and rapid adsorption for both metal ions. The relatively high uptake of the metal ions at the initial contact time could be due to the availability of a larger surface area of the adsorbent^53^. However, as the adsorption sites became saturated, the adsorption rate gradually diminished over time until it stabilized. The residual Pb(II) ions concentration was 37% at 60 min, 22% at 90 min, 15% at 120 min, 10% at 150 min, and 8.45% at 180 min. While the residual Cr(VI) ions concentration was 15% at 60 min, 13.7% at 120 min, 11.85% at 150 min, and 11.35% at 180 min of contact time. Hence, the APS adsorbent removed about 89% of Cr(VI) ions and about 91.55% of Pb(II) ions at 180 min of contact time, after which there was no significant increase in the removal efficiency. The slower uptake of the ions between 150 and 180 min could be due to the formation of metal ions monolayer on the adsorbent surface which slows down the adsorption rate^54^. Therefore, 180 min was adopted as the steady-state equilibrium condition, and other experiments were carried out at that period.

#### Effect of temperature

**Figure 8:**
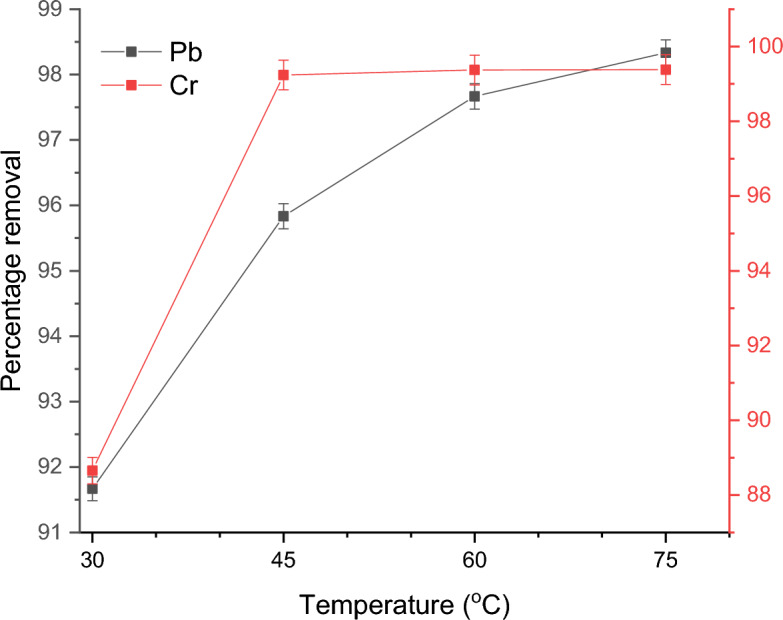
Effect of temperature on the adsorption of Pb(II) and Cr(VI) ions onto the APS.

Figure 8 shows the effect of adsorption temperature of 30, 45, 60, or 75 °C on the removal of Pb(II) and Cr(VI) ions by the APS at an adsorbent dosage of 1.5 g and contact time of 180 min. The adsorption of the metal ions increased with rising temperature values from 30 to 75 °C for Pb(II) ions. This is a typical effect of temperature on the adsorption process since the rise in temperature increases the rate of adsorbate molecule diffusion across the interior pores and external boundary layer of adsorbent particles^53^. However, adsorption equilibrium temperature was achieved with Cr(VI) ions at 45 °C. This could be due to the limited mass transfer of the adsorbate molecules from the bulk liquid to the external surface of the adsorbent and consequently lead to the attainment of equilibrium at a lower temperature of 45 ^o^C^55^. At the highest temperature of 75 °C, it was feasible to remove Pb(II) and Cr(VI) ions with approximately 99% removal efficiency using 1.5 g adsorbent dosage. The capacity of the adsorbent to bind more heavy metals as the temperature rises is a sign of endothermic adsorption.

For the elimination of Pb(II) ions, Nwoko et al.^56^ reported comparable results when maize and plantain stalks were utilized as adsorbents. Jacob et al.^57^ also discovered a rise in the percentage removal of Cr(III) ions to 86% when the temperature increased from 20 ^o^C to 40 °C.

### Adsorption kinetics

To learn more about the performance of APS adsorbent in the adsorption of Pb(II) and Cr(VI) ions from electroplating effluent, pseudo-first-order (Eq. 3), pseudo-second-order (Eq. 4), and intra-particle diffusion (Eq. 5) models of kinetics were used to fit the experimental data. The kinetic parameters for the adsorption process were assessed for contact times ranging from 30 to 180 min, while the quantity of heavy metals removed by the adsorbent during this time was monitored.

3 $$ln\left({q}_{e}-{q}_{t}\right)=ln{q}_{e}-{k}_{1}t$$

4 $$\frac{t}{{q}_{t}}=\frac{1}{h}+\frac{1}{{q}_{e}}t$$

5 $${q}_{t}={k}_{diff}{t}^\frac{1}{2}+C$$

where q_e_ and q_t_ represent the adsorption capacities at equilibrium and at time t, respectively, measured in mg/g. The rate constant for pseudo-first-order adsorption is denoted as k_1_ (1/min), and h is calculated as k_2_q_e__2_ (mg/g.min), where k_2_ represents the rate constant for pseudo-second-order adsorption (1/mg.min). Additionally, q_t_ represents the amount of heavy metal adsorbed in mg/g at time t, and k_diff_ (mg/g·min) represents the rate constant for intra-particle diffusion. The value of C provides information about the thickness of the boundary layer effect.

**Figure 9:**
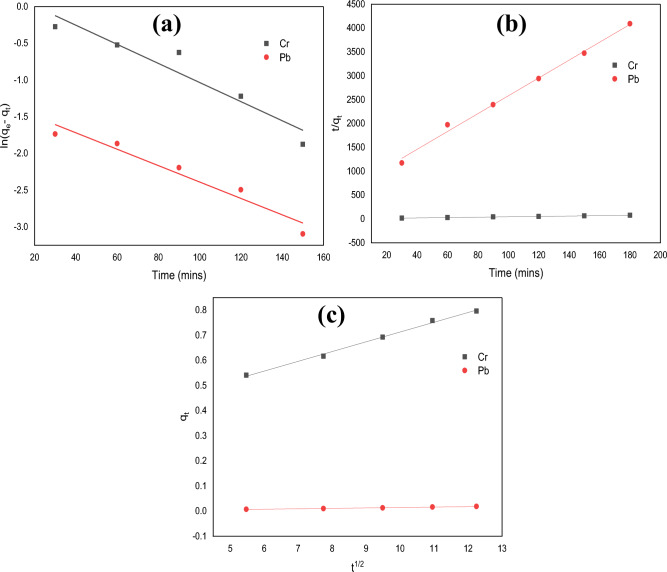
Adsorption kinetics of Cr(VI) and Pb(II) ion by (**a**) pseudo-first-order, (**b**) pseudo-second-order, and (**c**) intraparticle diffusion model.

Figure 9(a–c) shows the experimental data for the removal of Pb(II) and Cr(VI) ions from the wastewater by the APS adsorbent which are fitted using models of pseudo-first-order, pseudo-second-order, and, intraparticle diffusion, respectively. The regression analysis with high R^2^ values as obtained from the figure and shown in Table 3 reveals that the models fitted well with the experimental data. Generally, a model is successful in characterising the kinetics of the adsorption process if the R^2^ value is relatively high (close to 1). Overall, all the three models are applicable in the description of the adsorption process as shown by the relatively high R^2^ values in Table 3. This is an indication that the adsorption process could involve a combination of the direct proportion between the rate of adsorption and the adsorbate concentration (pseudo-first-order) (Fig. 9a). Also, it could be the direct proportion between the rate of adsorption and the square of the adsorbate concentration (pseudo-second-order) (Fig. 9b). The diffusion of adsorbate molecules within the pores of the adsorbent material (intraparticle diffusion) (Fig. 9c) could also describe the process.

**Table 3:** Calculated data for Pb(II) and Cr(VI) ions adsorption kinetics from pseudo-first order, pseudo-second order, and intra-particle diffusion models.

Metal ions	Pseudo-first-order kinetic model			Pseudo-second-order kinetic model			Intra-particle diffusion Model		
	q_e_ (mg/g)	k_1_ (1/min)	R^2^	q_e_ (mg/g)	k_2_ (mg g/min)	R^2^	C	K_diff_ (mg/g min)	R^2^
Pb	0.055	0.0258	0.928	0.0535	0.492633	0.998	0.0124	0.0028	0.956
Cr	1.850	0.0399	0.887	2.5242	0.02879	0.992	1.4701	0.0697	0.949

Typically, regression correlation coefficient values of < 0.5 could not be used to deduce that there is a positive fitting of the experimental data to the model^53^. In addition, since the estimated and experimental q_e_ values are comparable and have large R^2^ values (all > 0.9), it suggests that chemisorption is the most plausible adsorption mechanism of Pb(II) and Cr(VI) ions onto the APS adsorbent. These findings correlate with the findings of Obi and Njoku^37^ who reported that Pb(II) ions sorption in an aqueous solution followed the second order, reporting correlation values (R^2^) of 0.991 for Pb(II) ion. Similarly, Garg et al.^50^ also reported that the pseudo-second-order kinetics model could be used to explain the adsorption of Cr(VI) ions using corncob to treat electroplating wastewater. For the intra-particle diffusion model, both the linear and equilibrium intervals have high R^2^ values. The adsorption of Pb(II) and Cr(VI) ions by the APS adsorbent may therefore include an intra-particle diffusion process. However, the fact that the C value is > 0 demonstrates that the rate of the adsorption process is not set by intra-particle diffusion.

### Adsorption isotherms

**Figure 10:**
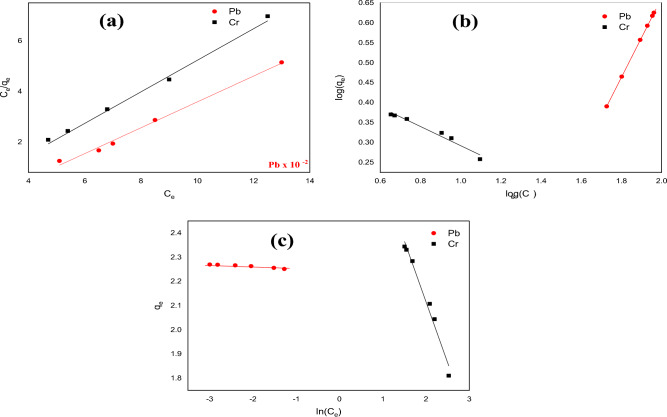
(**a**) Langmuir isotherm, (**b**) Freundlich isotherm, and (**c**) Temkin isotherm for the adsorption of Pb(II) and Cr(VI) ions on APS.

The adsorption of Pb(II) and Cr(VI) ions by the APS adsorbent was further studied using well-known adsorption isotherm models to identify the adsorption mechanism. The Langmuir (Eq. 6), Freundlich (Eq. 7), and Temkin isotherm (Eq. 8) equations were employed to identify the type of adsorption that occurred in this study. Figure 10(a-c) illustrates the plots of the Langmuir, Freundlich, and Temkin isotherms, respectively, using the linearized forms of the equations.

6a $$\frac{{C}_{e}}{{q}_{e}}=\frac{1}{{K}_{L}*{q}_{max}}+\frac{{C}_{e}}{{q}_{max}}$$

6b $${R}_{L}=\frac{1}{{1+K}_{L}{C}_{o}}$$

7 $$log{q}_{e}=log{K}_{f}+\frac{1}{n}log{C}_{e}$$

8 $${q}_{e}={B}_{T}ln\left({C}_{e}\right)+{B}_{T}ln{K}_{T}$$

where ${q}_{e}$ is the adsorption amount at equilibrium (mg/g), K_L_ is the Langmuir constant (L/mg), ${C}_{e}$ is the equilibrium concentration of Cr(VI) and Pb(II) ions (mg/L), $\frac{1}{n}$ is the Freundlich constant, which indicates the adsorption intensity, ${K}_{f}$ is the adsorption coefficient, ${B}_{T}$ and ${K}_{T}$ are the Temkin isotherm constants.

The calculated data obtained for the three isotherms investigated in this study are listed in Table 4. Also, Fig. 10a indicates that the amount of APS adsorbent that adsorbs the two metals ions increased with concentration. The Langmuir fitting coefficient of APS for Pb(II) and Cr(VI) ions was higher (R^2^ > 0.99), indicating that the adsorption process was essentially monolayer in nature since the majority of adsorbed compounds were evenly distributed over the adsorbent surface to achieve adsorption saturation^15^. The value of R_L_ which is 0.999 and 0.038 for Pb(II) and Cr(VI) ions, respectively, is used to identify whether the isotherm is irreversible (R_L_ = 0), favourable (0 < R_L_ < 1), linear (R_L_ = 1), or unfavourable (R_L_ > 1)^10^. The value of R_L_ obtained in this experiment for both the Pb(II) and Cr(VI) ions falls within a range of 0 < R_L_ < 1, which indicated that the two metal ions were effectively adsorbed onto the APS.

**Table 4:** Calculated values of constants of Langmuir, Freundlich and Temkin isotherms.

Metal ions	Langmuir isotherm				Freundlich isotherm			Temkin isotherm		
	q_max_	K_.L_	R_.L_	R^2^	${K}_{f}$	N	R^2^	${B}_{T}$(kJ/mol)	${K}_{T}$	R^2^
Pb(II)	0.024	8.91E−05	0.999	0.978	3.407	− 4.16	0.960	− 0.011	0.258	0.943
Cr(VI)	1.63	6.36E−01	0.038	0.993	0.001	2	1	− 0.503	0.002	0.975

The ability of the Freundlich model to fit the experimental data was evaluated using the plot of log ${q}_{e}$ against log ${C}_{e}$ shown in Fig. 10b. The values of ${K}_{f}$ and n were obtained, respectively, using the intercept and slope of the plot. The plots resulted in graphs with an R^2^ value of almost 1, indicating a very high precision, however, the value of n is less than 1. Whereas, the value of n is required to be greater than 1 for favourable adsorption^58^. Therefore, the adsorption process did not follow the Freundlich model.

The Temkin isotherm model was also investigated to appraise the adsorption potentials of the adsorbent (APS) for adsorbates (Cr(VI) and Pb(II) ions). This was done by having a straight line upon plotting ln ${C}_{e}$ vs ${q}_{e}$ in the Temkin isotherm (Eq. 8) and displayed in Fig. 10c. The Temkin isotherm parameters obtained from the analysis of the plot are also given in Table 4.

Where ${B}_{T}$ is the index for the heat of adsorption for APS adsorbent (kJ/mole) and ${K}_{T}$ is the bond constant that represents the maximum binding energy (L/g)^59^.

As shown in the table, ${B}_{T}$ values are − 0.011 kJ/mol and − 0.503 kJ/mol for Pb(II) and Cr(VI) ions, respectively. The low ${B}_{T}$ values obtained designate a weak interaction between adsorbate (Pb(II) and Cr(VI) ions) and adsorbent (APS) which support an ion-exchange mechanism in this study^60^. Also, the negative ${B}_{T}$ values obtained suggest that the adsorption process is endothermic. Furthermore, the ${K}_{T}$ values of 0.258 L/g and 0.002 L/g were obtained for Pb(II) and Cr(VI) ions, respectively. The low ${K}_{T}$ values indicate a lower APS-metal ion potential for Pb(II) and Cr(VI) ions, possibly due to their small ionic radii^60^. Also, the R^2^ of 0.943 and 0.975 were obtained for Pb(II) and Cr(VI) ions, respectively (Table 4), indicating that the Temkin model fits well with the equilibrium data for the adsorption process^8^.

### Thermodynamics of the adsorption process

**Figure 11:**
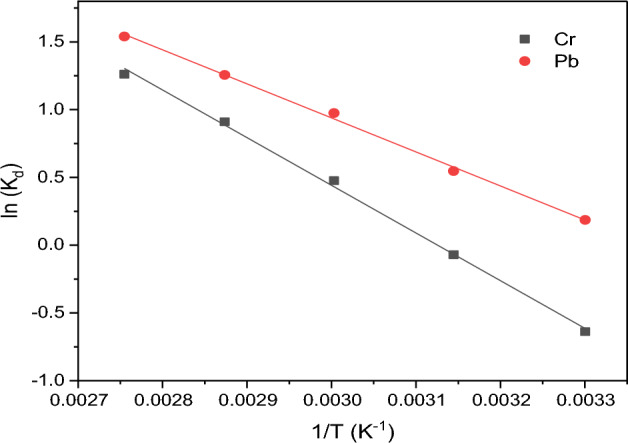
Thermodynamic plots of Cr(VI) and Pb(II) ions adsorption by APS adsorbent.

Thermodynamic investigations are crucial in revealing the energetics of the adsorption process (exothermic or endothermic). Also, the obtained data can be used to determine whether the system is at equilibrium or not and predict the feasibility of a chemical reaction. The standard enthalpy (ΔH) and standard entropy (ΔS) were determined from the plot of ln (K_d_) against 1/T in the thermodynamics studies of Pb(II) and Cr(VI) ions by the APS adsorbent which is shown in Fig. 11. A summary of the calculated thermodynamic parameters for the adsorption of Pb(II) and Cr(VI) ions by the APS adsorbent are shown in Table 5. The Gibbs free energy (ΔG) which measures the spontaneity of the adsorption process indicates that a higher negative value denotes a scenario that is more energy-beneficial (spontaneous), while a positive value suggests non-spontaneous. Thus, the standard ΔG^o^ values for Pb(II) and Cr(VI) ions decrease as the temperature rises, which indicates that the adsorption process was spontaneous. Kumar^62^ highlighted that the negative value of ΔG^o^ affirms both the efficacy of the adsorption process and its spontaneity. Since the adsorption of both Pb(II) and Cr(VI) ions exhibited negative values of ΔG^o^, it implies that the adsorption process is favourable and spontaneous^58^. The positive values of ΔH for the adsorption of the two metal ions confirm that the process is endothermic as suggested by the Temkin isotherm analysis. This requires energy to facilitate the formation of bonds between the ions and the functional groups of the adsorbent^63^. This ensures that when the ions bind to the adsorbent, weak bonds can be formed, and the repelling force of attraction can be overpowered. Meanwhile, the positive entropy values suggest an increase in randomness in the solid–liquid interface or solution, which was caused by the affinity of the ions for the adsorbent. This indicates that the removal of the two metal ions was reversible and that the energy of the adsorption process is constant^64^. Furthermore, the results demonstrated that agricultural waste-derived adsorbents can spontaneously bind metal ions in a stable and thermodynamically favourable manner^65^.

**Table 5:** Thermodynamic parameters for the adsorption of Pb and Cr by APS.

Metals ions	ΔH (kJ/mol)	ΔS (kJ/mol K)	ΔG (kJ/mol) @303	ΔG (kJ/mol) @ 318 K	ΔG (kJ/mol) @ 333 K	ΔG (kJ/mol) @ 348	R^2^
Pb(II)	76.272	0.291	− 11.9205	− 16.29	− 20.65	− 25.018	0.9975
Cr(VI)	128.11	0.469	− 13.8698	− 20.90	− 27.93	− 34.96	0.9978

## Reusability and regeneration studies

The reusability and regenerative attributes are essential properties of an adsorbent as they govern the economic feasibility of the adsorption process^6^. The reusability of adsorbent also reduces secondary pollution and the cost of production by utilising one adsorbent for multiple cycles^61^. In this study, the APS reusability was studied over eight cycles with the same adsorbent when the operating conditions were maintained at the adsorbent dosage of 1.5 g, contact time of 180 min and temperature of 75 °C. After each circle of the experiment, the spent adsorbent was retrieved through filtration, washed several times with de-ionised water and oven-dried at 100 °C until constant weight was achieved. The residual concentrations of the Pb(II) and Cr(VI) ions in the treated wastewater were measured using the AAS and the percentage removal was evaluated using Eq. (1).

**Figure 12:**
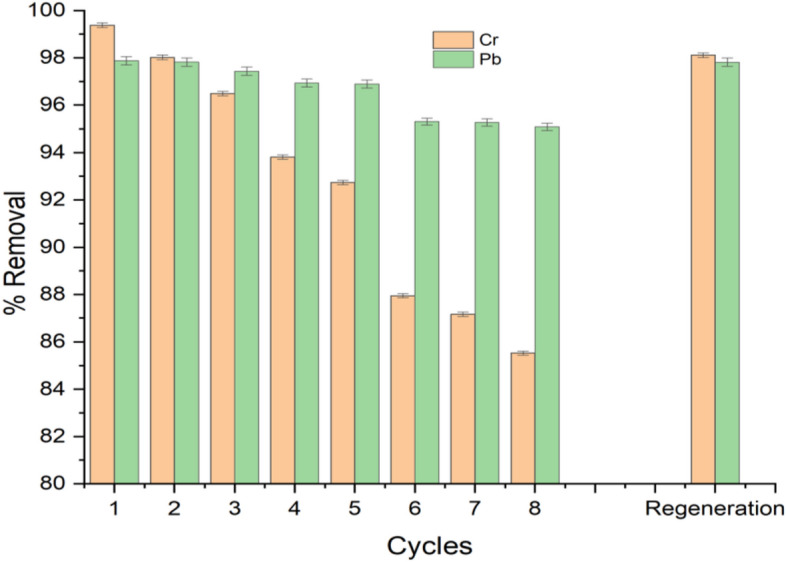
Reusability and regeneration of APS for removal of Pb(II) and Cr(VI) ions from the wastewater.

The estimated percentage removal are 98.33% and 99.38% (cycle 1), 98.00% and 98.02% (cycle 2), 95.89% and 96.49 (cycle 3), 93.11% and 93.81% (cycle 4) for Pb(II) and Cr(VI) ions respectively, as shown in Fig. 12. These results revealed that the APS adsorbent could be envisaged to have good reusability and stability properties^6^. However, there was a reduced percentage removal of the ions from 5th cycle to 8th cycle compared to the removal efficiency of the adsorbent for the first 4 cycles. This shows that the capacity of adsorbent gradually decreases with each cycle which is similar to the findings in the literature^8^. Thus, the APS adsorbent showed high stability for the first 4 cycles of the study and achieved high removal percentages for the Pb(II) and Cr(VI) ions with a noticeable decrease in the efficiency from 5th to 8th cycle. This reduction can be attributed to the deposition of metal ion particles on the surface of APS, which resulted in the blockage of adsorbent pores and finally decreased the adsorption capacity^51^. After the 8th cycle, the adsorbent was subsequently regenerated by eluting the ions in it with 0.1 M NaOH, this is because alkalis were reported to be the best desorbing agents for cellulose-based adsorbents^61^. Thereafter, the adsorbent was rinsed with deionised water until the pH was neutral and the sample was oven-dried at 100 ^o^C for 1 h, to complete the regeneration process. After the regeneration, the adsorbent was further used to treat the effluent and the percentage removal obtained was 97.97% and 98.11% for Pb(II) and Cr(VI) ions respectively. These results are closer to the percentage removal obtained for cycle 1 (98.33% and 99.38% for Pb(II) and Cr(VI) ions respectively). This implies that the regeneration of APS is an excellent reusable and easily regenerated adsorbent for the removal of both Pb(II) and Cr(VI) ions from the electroplating wastewater.

## Conclusion

The study set out to probe the production and characterisation of APS adsorbent from RPS and aluminium trash cans, as well as their adsorptive removal of metal ions from electroplating wastewater. The RPS and aluminium trash cans were effectively converted into the APS adsorbent, which offered distinct advantages such as quick preparation, and strong adsorptive capacity. The characterisation studies concluded that the APS has a larger surface area and pore spaces for the uptake of the metal ions than the RPS. The presence of the functional groups (hydroxyl, carboxylic, and alkyl groups) played significant roles in the adsorption of the Pb(II) and Cr(VI) ions from the wastewater. The highest percentage removal of Pb(II) and Cr(VI) ions by the APS was observed at 1.5 g dosage, 180 min, and 75 °C, which was 98.33% and 99.38%, respectively. The study also concluded that the APS adsorbent is an excellent reusable and easily regenerated adsorbent for the removal of both Pb(II) and Cr(VI) ions from the electroplating wastewater.

Therefore, the APS adsorbent was sufficiently used for the sequestration of Pb(II) and Cr(VI) ions in the electroplating wastewater. Future studies could investigate the use of this adsorbent for the removal of other heavy metal ions in different sources of wastewater.

## Data Availability

All data generated or analyzed during this study are included in this published article.
